# Supplementary material for: Elevated Extracellular HSP72 and Blunted Heat Shock Response in Severe COVID-19 Patients
Source: Biomolecules. 2022 Sep 26;12(10):1374. doi: 10.3390/biom12101374 (PMC9599720; doi:10.3390/biom12101374)
Supplement: Supplementary file 1 [file biomolecules-12-01374-s001.zip › biomolecules-1906451-supplementary.pdf]

Table S1. Patient Medication Profile

	Control		Diabetes	
**Medications**	n	%	n	%
**ACE inhibitors**	1	3,8	10	33,3
**Metformin**	0	0	15	51,7
**Sulfonylurea**	0	0	5	17,2
**DPP4 inhibitors**	0	0	1	3,4
**Insulin NPH**	0	0	5	16,7
**Regular Insulin**	0	0	1	3,3
**Statins**	2	7,7	10	34,5

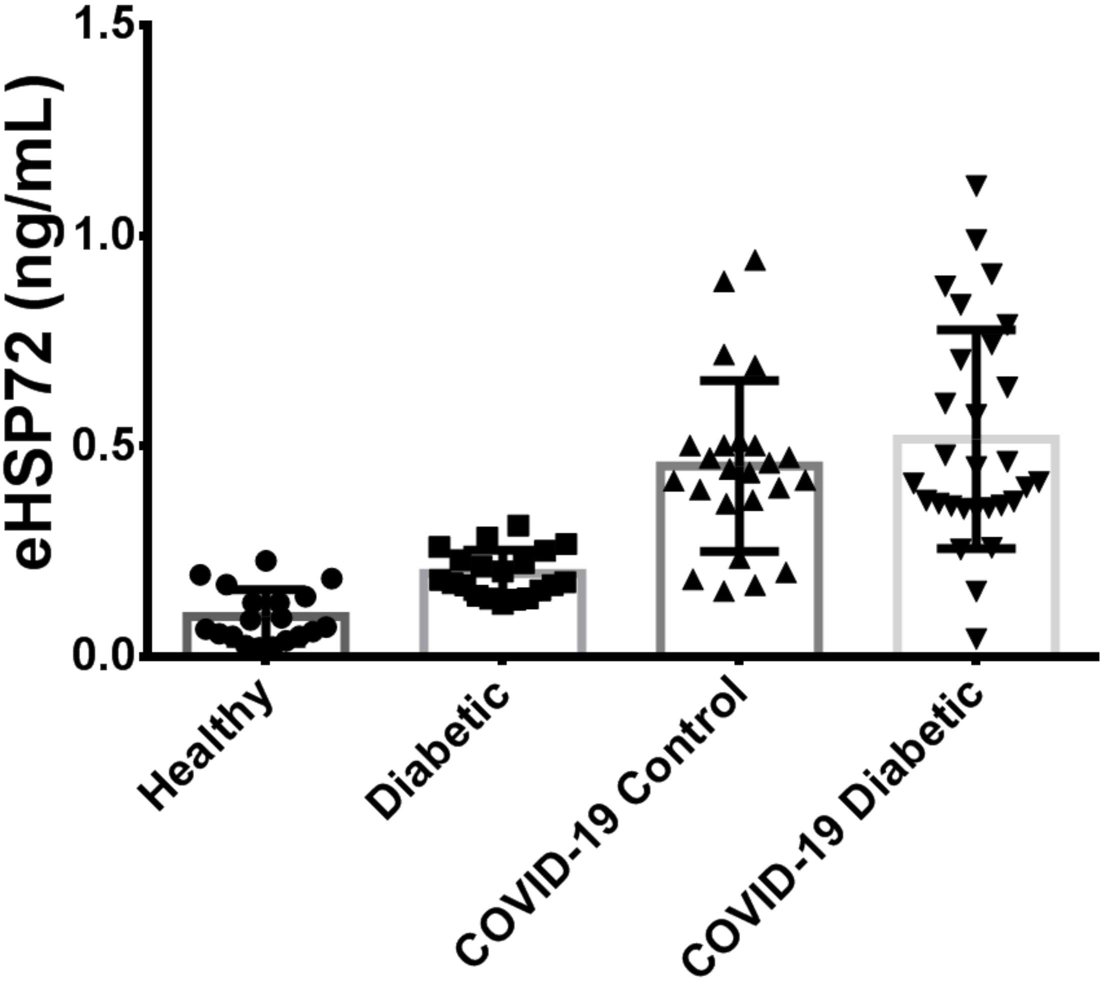

Figure S1. eHSP72 with separate groups
